# Epigenetic Influences in the Obesity/Colorectal Cancer Axis: A Novel Theragnostic Avenue

**DOI:** 10.1155/2019/7406078

**Published:** 2019-03-17

**Authors:** Duncan Ayers, Hatim Boughanem, Manuel Macías-González

**Affiliations:** ^1^Centre for Molecular Medicine and Biobanking, University of Malta, Malta; ^2^Faculty of Biology, Medicine and Health, University of Manchester, UK; ^3^Instituto de Investigación Biomédica de Málaga (IBIMA), Faculty of Science, University of Malaga, Málaga, Spain; ^4^Unidad de Gestión Clínica de Endocrinología y Nutrición del Hospital Virgen de la Victoria, Instituto de Investigación Biomédica de Málaga (IBIMA), Universidad de Málaga, Málaga, Spain; ^5^CIBER Fisiopatología de la Obesidad y Nutrición (CB06/03), Madrid, Spain

## Abstract

The World Health Organization (WHO) considers that obesity has reached proportions of pandemic. Experts also insist on the importance of considering obesity as a chronic disease and one of the main contributors to the worldwide burden of other nontransmissible chronic diseases, which have a great impact on health, lifestyle, and economic cost. One of the most current challenges of biomedical science faces is to understand the origin of the chronic nontransmissible diseases, such as obesity and cancer. There is a large evidence, both in epidemiological studies in humans and in animal models, of the association between obesity and an increased risk of cancer incidence. In the last years, the initial discovery of epigenetic mechanisms represents the most relevant finding to explain how the genome interacts with environmental factors and the ripple effects on disease pathogeneses. Since then, all epigenetic process has been investigated by the scientific communities for nearly two decades to determine which components are involved in this process. DNA/RNA methylation and miRNA are classified as two of the most important representative classes of such epigenetic mechanisms and dysregulated activity of such mechanism can certainly contribute to disease pathogenesis and/or progression especially in tumors. This review article serves to highlight the impact of DNA/RNA methylation and miRNA-based epigenetic mechanism activities in the interplay between obesity and the development and clinical significance of colorectal cancer.

## 1. Introduction: Background and Clinical Importance

The World Health Organization (WHO) considers that obesity has reached pandemic proportions: more than 1900 million adults are overweight and, of these over 650 million of them, obesity [1]. Epidemiological experts also insist that obesity must be considered as a chronic disease and one of the main contributors to the worldwide burdens of other nontransmissible chronic diseases, such as autoimmune, inflammatory, neurodegenerative, and cardiovascular diseases, including diabetes or cancer [2, 3]. One of the most relevant challenges that biomedical science is trying to solve is finding the pathogenic mechanism of chronic noncommunicable diseases of metabolic origin, such as obesity and cancer. There is a large evidence of the linking between obesity and cancer. This link has also been supported by animal experiments, where obesity and cancer have been modified by dietary types [4]. Indeed, a strong relationship has been observed between adiposity and the risk of suffering from up to 13 different types of cancer, although there is a substantial heterogeneity between the different studies [5, 6].

**Figure 1:**
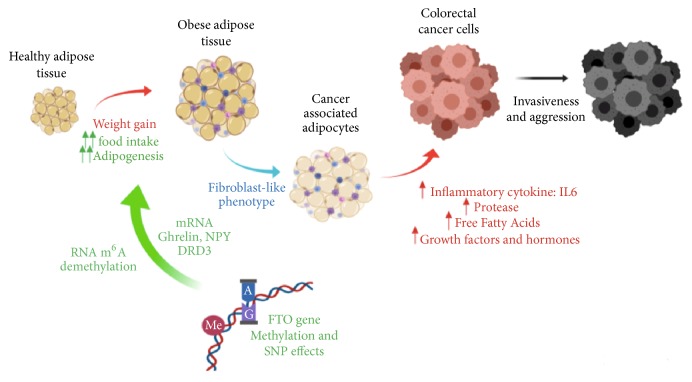
Proposed mechanisms linking FTO gene, obesity, and cancer. The weight gain, malfunctioning of the FTO gene leading to increase food intake and adipogenesis process could develop obesity, especially abdominal obesity. It is also linked to adipocyte hypertrophy and hypoxia. The hypertrophied adipose tissue acquires endocrine characteristics like fibroblasts, which produce an increase of adipokine and hormone secretion profile, proteases, and free fatty acids that may promote the stimulation of a microenvironment favorable for not only tumorigenesis, but acquire new properties as invasiveness and aggression. Abbreviations: m6A: N6-Methyladenosine; NPY: Neuropeptide Y; DRD3: Dopamine Receptor type D3; FTO: Fat-mass and obesity-associated; SNP: Single-nucleotide polymorphism; IL6: Interleukin 6.

During tumorigenesis, adipocytes that are found near to cancer cells suffer several morphological and biochemical alterations and are implicated in developing of the Cancer-Associated Adipocytes (CAAs) which influence cancer cell malignancy. CAAs located close to the invasive front acquire different fibroblast-like features. Lipids secreted by adipocytes are transferred to cancer cells and used for energy production through beta-oxidation. The loss of expression of differentiation markers in CAAs such as adiponectin or leptin and the increased secretion of proinflammatory cytokines as Interleukin 6 (IL-6) and tumor necrosis factor (TNF) generate a permissive niche for tumor growth and dissemination by stimulating adhesion, migration, and invasion proprieties of malignant cells (Figure 1). Moreover, the rapid expansion of adipose tissue produces oxygen deficiency and promotes angiogenesis improving the tumor spreading [7, 8].

In particular, the new location of the CAAs transforms them in highly metabolic cell that secrete greater amounts of cytokines associated with insulin resistance [9]. In any case, the ectopic fat depots were mostly associated with paracrine effects in the tumor microenvironment. The ectopic local adiposity corresponds to inflammation and was mainly associated with colon and pancreatic cancer, breast tumorigenesis, and hepatocellular carcinoma [10, 11]. On the other hand, the systemic ectopic fat, also known as “central adiposity”, corresponds to visceral adipose tissue (VAT), is responsible for altered levels of sex steroids, insulin resistance, and chronic inflammation, and increased risk of colorectal neoplasia [12, 13].

## 2. Epigenetics and Epitranscriptomics Mechanism of* FTO* Gene in Obesity and Colorectal Cancer: A Potential Biomarker

Epigenetic processes, including DNA methylation, modification of histones and noncoding RNAs of different size and function, change genes expression without modifying the DNA sequence. They are sensitive to external (e.g., diet and physical activity), internal (hormones and inflammation markers), and genetic factors and reversible and can be passed on to later generations. Two large-scale epigenetic studies have identified a large number of DNA methylation loci associated with Body Mass Index (BMI) [14]. Clearly, more evidence is needed to determine a role (causal) of epigenetic processes in obesity but assessing the epigenome at the right time in life and in the relevant tissues is an important barrier for human studies [14]. Recent studies in genetic association analyses show that alterations in DNA methylation are predominantly the consequence of adiposity, rather than the cause [15].

Different polymorphisms of the* FTO* (fat-mass and obesity-associated) gene have been consistently associated with obesity. The great success on a large scale of the genome-wide association studies (GWAS) was the discovery that the* FTO* locus could regulate the expression of several genes despite their distance in the linear sequence of DNA to influence body weight, through the regulation of epigenetic mechanisms related to obesity [16]. However, the molecular mechanisms responsible for the effect of* FTO* gene on obesity are not known yet. Recent genome studies reveal that genetic variants in this gene are associated not only with human adiposity and metabolic disorders, but also with several cancers including breast cancer, endometrial cancer, pancreatic cancer, and colorectal cancer, since it can activate several signaling and hormonal pathways to increase cancer incidence. These hormones include ghrelin, neuropeptide Y, oxytocin, and leptin. Therefore, the FTO polymorphisms could exert an influence on hormonal balance and physiologic factors and might increase cancer risk (Figure 1) [17–21]. However, the link between FTO polymorphism, hormonal metabolism, and cancer risk is yet uncertain.

Epigenomics connects genomics with environmental factors in the pathogenesis of several diseases. In fact, another emerging concept in the regulation of gene expression is that some modified nucleotides are found internally in mRNA. These modifications constitute a new concept of epitranscriptomic code. The initial concept of epitranscriptome was introduced with the transcriptomic scale mapping of N6-methyladenosine (m^6^A), which revealed that it is found in at least 25% of all mRNAs, typically near stop codons [22]. In fact, the most significant advance on* FTO* gene research is the recent discovery of FTO protein as the first m^6^A RNA demethylase [23]. This finding provides solid evidence that the dynamic and reversible enzymatic modification of m^6^A in RNA can act as a new epitranscriptomic marker [24]. Recent and consistent studies show that the biological functions of a number of cancers can be regulated at the epitranscriptional level, indicating the pharmaceutical potential of these studies. Indeed, m^6^A process has been associated with cancer, contributing to the self-renewal of cancer stem cell, promotion of cancer cell proliferation, and resistance to radiotherapy or chemotherapy, and playing a crucial role in the metabolism of mRNA and regulation of noncoding RNAs in cancer cells [25].

## 3. Experimental Framework of Current Research: DNA Methylation in Obesity and Colorectal Cancer

Sometimes, it is necessary to go beyond the sequences of the genes to understand better the regulatory mechanisms of their expression, because, often, genomic studies do not identify the causes and etiologies of many diseases. One of the important mechanisms that explain the regulatory elements of the genes is the epigenetic modifications in DNA such as methylation, which consist in the addition of a methyl group to position 5 of the cytosine bases, which generates 5-methylcytosine (m^5^C). This process can take place passively, without methylation of the newly synthesized chain after DNA replication, or actively, by mechanisms that do not depend on replication. Active demethylation of DNA has just begun to be studied, along with the discovery of another methylated variant of cytosine, 5-hydroxymethylcytosine (hm^5^C), and the enzymes that catalyze its generation from m^5^C [26]. There is currently an adequate level of understanding about m^5^C and its impact on gene expression, but the products of DNA demethylation are not known in detail, whether they are only intermediates or epigenetic marks by themselves. Therefore, their study will reveal in a novel way the implication of epigenetics in cancer and obesity processes.

There have been published some studies that show abnormal distributions of differentially methylated regions (DMR) overlap, such as CpG hypermethylated islands, which may explain the epigenetic instability that drives the onset of cancer in individuals with obesity [27]. These studies identify a potential epigenome mark of obesity related to breast and colorectal cancer that could be useful for precision medicine in the management of these diseases considering adiposity as a relevant risk factor [28]. These results also show that the molecular heterogeneity of colorectal cancer can be influenced by modifiable risk factors such as obesity. Strikingly, by performing a comprehensive GWAS of the DNA methylation that interrogates 485000 CpG sites, it has been demonstrated for the first time the existence of a specific methylome profile in colorectal cancer associated with excess of adiposity. The epigenome-wide analysis identified 46 unique genes that exhibited differential methylation in the promoter region and island or shore, which could be established as an epigenetic signature of obesity-related colorectal cancer [28].

Epigenetic gene inactivation in transformed cells involves many “belts of silencing”. One of the best-known lesions of the malignant cell is the transcriptional repression of tumor-suppressor genes by promoter CpG island hypermethylation. Currently, the scientific community are beginning to understand a great deal of epigenetic silencing of tumor-suppressor genes in human cancer by CpG island promoter hypermethylation. In the process of completing the molecular dissection of the entire epigenetic machinery involved in methylation-associated silencing, such as DNA methyltransferases, methyl-CpG binding domain proteins, histone deacetylases, histone methyltransferases, histone demethylases, and specially the identification of many more hypermethylation-silenced miRNA genes with tumor-suppressor function in human cancer, the epigenetic silencing of these miRNAs will become an excellent target [29]. The importance of hypermethylation events is already in evidence at the bedside of cancer patients in the form of cancer detection markers and chemotherapy predictors, and in the approval of epigenetic drugs for the treatment of hematological malignancies. In the very near future, the synergy of candidate gene approaches and large-scale epigenomic technologies will yield the complete DNA hypermethylome of cancer cells. In fact, it has been recently published that adipose tissue may be a key factor in colorectal cancer development [29].

The low vitamin D levels in colorectal cancer and high expression of vitamin D receptor in adipose tissue may, at least in part, mediate this relationship by modifying adipose tissue DNA methylation and promoting inflammation. Although more studies are needed to discover the precise mediators and mechanisms that determine this relationship, the possible mediation of adipose tissue in colorectal cancer should be born in mind to create new treatments and preventive strategies for colorectal cancer [30, 31].

The recent concept of the implication of epigenetic variations in numerous pathologies has directed attention towards the development of new markers for its detection. Therefore, it has become necessary to develop new analytical tests to identify epigenetic changes through molecular biomarkers related to the active or silenced state of the regions in which they are found, in order to detect changes in methylation, both in DNA and in RNA, whose manifestations can be revealed in advance even of the development of the disease. Every cell, including small and early cancerous lesions, leaves a record of its physiological state that is reflected in the fragments of DNA and RNA, proteomic and circulating metabolic products. The molecules in circulation make up a file of biomarkers, which constitute a potential reservoir of diagnostic information for the complete organism, whose clinical use depends on the sensitivity of the detection and quantification methods of methylated DNA/RNA from liquid biopsies [32, 33]. Despite the urgent clinical need, the number of epigenetic biomarkers developed so far is really low. Given its stability, the methylation of the CpG islands constitutes a very favorable epigenetic biomarker, which allows designing methods to detect the signals of methylated DNA from specific regions of the genome, usually not methylated. However, the understanding of epigenetic factors may play a role in the search for new biomarkers, necessary to define obesity and cancer in a more precise way [33].

## 4. MicroRNAs: Understanding the Mechanism of Action

The MicroRNAs (miRNAs) consist of the most clinically important noncoding RNA families and have been investigated by the scientific communities for nearly two decades, since the initial discovery of the RNA interference (RNAi) pathway by Fire and Mello in 1998 [34]. Structurally, once within the cytoplasm each miRNA consists of a single RNA strand, self-folded into a duplex, with one of the duplex ends containing the active (mature) 19-22 nucleotide-long miRNA sequence [35]. Mature miRNAs, once incorporated into the RNAi machinery, act as agents of posttranscriptional gene regulators by binding with near-complementarity to the 3′-UTR of their designated target transcripts with consequent inhibition of ribosomal activity that typically occurs during the process of translation [35].

This regulatory function by miRNAs revealed a further level of complexity in the nature of gene regulation within a vast spectrum of physiological processes and, consequently, highlighted the influence of miRNAs in human disease pathology and progression and/or inhibition, whenever such miRNAs exhibit dysregulated activity [36]. In addition, the sheer fact that there exist over 2600 validated miRNAs in humans is a reminder of the intricate complexities underlying gene regulatory processes, where the dysregulated expression of an individual miRNA or a whole network of miRNAs could contribute to disease pathogenesis and/or progression, especially in tumors [37].

## 5. miRNA Influences in Obesity

The first indications of miRNA involvement in the regulation of lipid metabolism date back to 2006, where the study conducted by Esau and colleagues elucidated that miR-122 upregulation led to increased plasma cholesterol levels, together with a decrease in lipid metabolic activities, in murine study models [38]. Furthermore, the study carried out by Takanabe and colleagues in 2008 provided the first breakthrough to identify a direct association of miRNA dysregulated activity with obesity [39]. In this study, miR-143 was recognized to be upregulated within mesenteric fatty tissues of mice undergoing a high-fat diet and was also correlated with plasma leptin levels, with leptin being already established as a major adipocytokine [39]. Other initial discoveries to confirm the link between miRNA dysregulation and obesity include the study performed by Xie and colleagues in 2009 [40]. The study involved the employment of miRNA microarrays to analyze differing miRNA expression profiles for over 370 miRNAs during the adipogenesis of adipocytes and preadipocyte 3T3-L1 cells deriving from leptin deficient ob/ob, diet-induced obese mice [40].

The conclusions of this seminal study were that miR-143 and miR-103 upregulation both lead to exacerbated adipogenesis, with additional evidence provided by the correlated increases in adipogenesis markers and triglyceride production in such murine in vivo models [40]. The first human study on the implication of miRNA activity in obesity was conducted in the same year by Kloting and colleagues [41]. In this particular study, multiple fat deposit samples were collected from overweight and obese patients and analyzed for a panel of 155 differing miRNAs using Real Time qPCR [41]. The overall results of this study revealed that the concomitant expression pattern of seven miRNAs (miR-17-5p, miR-132, miR-99a, miR-134, miR-181a, miR-145, and miR-197) is highly correlated with the main diagnostic features for obesity, including factors such as adipose tissue morphology and unique metabolic parameters [41].

The first reporting of the link between circulating miRNA expression and obesity was by the Wang study in 2013 [42]. The results of this investigation revealed that circulating miR-130b levels were correlated with obesity in murine in vivo obesity models and in obese human volunteers [42]. In addition, miR-130b is typically secreted by adipocytes during the process of adipogenesis, with miR-130b also having the capacity to downregulate the expression of key muscular target genes such as peroxisome proliferator-activated receptor *γ* coactivator 1 alpha (PPARGC1A), leading to a reduction in lipid oxygenation capacity within muscles [42].

In 2014, Shao and colleagues revealed the initial connection between miR-33 and* FTO* expression [43]. This study elucidated that miR-33 in chickens was encoded on intron 16 of the sterol regulatory binding transcription factor 2 (SREBF2) and that the utilization of miR-33 antagonists in primary chicken hepatocytes led to exacerbated FTO transcript expression levels, suggesting that* FTO* is a direct target gene for miR-33 activity [43].

In 2015, Chu and colleagues highlighted the influence of miR-181a on lipid regulatory activities [44]. The study revealed that miR-181a exerts its lipid regulatory function by directly targeting isocitrate dehydrogenase 1 (IDH1), one of the key metabolic enzymes of the tricarboxylic acid cycle and promotor of lipid synthesis genes [44]. Another seminal study conducted in the same year by Wagschal and colleagues focused on cholesterol/triglyceride homeostasis-regulating miRNAs [45]. This large study involved a GWAS of over 188,000 individuals for 69 miRNAs located in close proximity to single-nucleotide polymorphisms (SNPs) associated with hyperlipidemia-inducing genes [45]. The results of this study identified four miRNAs (miR-128-1, miR-148a, miR-130b, and miR-301b) to regulate the transport of cholesterol-lipoproteins, namely, low-density lipoprotein receptor and the ATP-binding cassette A1 (ABCA1) [45]. These findings were further confirmed for miR-148a and miR-128-1 through in vivo murine models of obesity by overexpression/antisense targeting of such miRNAs [45].

More recently, the study by O'Neill and colleagues revealed the utility of analyzing the circulating expression level of miR-758-3p as a means for discerning between obesity and metabolic syndrome [46]. Further analysis for this novel circulating miRNA obesity biomarker, involving miRNA mimics/antagonists transfected in liver hepatocellular carcinoma cell line (HepG2 cells), demonstrated that miR-758-3p acts directly on the cholesterol efflux regulatory protein/ATP-binding cassette transporter type A1 (CERP/ABCA1) protein expression levels in a concomitant manner [46]. Another recent study, conducted by Castaño and colleagues, successfully identified a unique plasma exosome-based miRNA expression profile for obesity within murine in vivo models for obesity [47]. The expression profile, which consists of miR-122, miR-192, miR-27a-3p, and miR-27b-3p, was also found to be linked to the induction of glucose intolerance and hepatic steatosis within such murine in vivo models [47].

## 6. miRNA Influences in Colorectal Cancer

The study described above, conducted by Kloting and colleagues, could also be deemed to shed the first rays of light on the influence of miRNAs in obesity and cancer interactions, since miR-17-5p would be later recognized by the scientific community to be a member of the most notorious oncogenic polycistronic miR cluster, namely, the miR-17-92 polycistronic cluster, with such members being highly upregulated in a wide spectrum of human cancer models [41, 48].

Focusing on colorectal cancer, the level of influence by miRNA dysregulated activity has also been widely reported by the scientific community in the last 15 years, ever since the first reporting by Michael and colleagues in 2003 [49]. Among the more recent publications focusing on this research niche, the study by Xu and colleagues analyzed mRNA and miRNA expression profile datasets to identify regulatory networks that specifically control colorectal cancer (CRC) tumorigenesis [50]. This integrated omics analytical approach highlighted a miRNA-mRNA regulatory network that played a pivotal role in orchestrating the mitogen-activated protein kinase (MAPK) signaling pathway and downstream target genes concerned with cell fate [50]. A similar study focusing on the MAPK signaling pathway and its orchestration by miRNAs identified 13 miRNA-mRNA direct associations, including Transforming Growth Factor Beta Receptor 1 (TGFBR1) affected by miR-6071 and miR-2117 [51]. Another study, conducted by Ruhl and colleagues, revealed that miRNA-451a was consistently upregulated following a single dose of 2 Gy or 10 Gy gamma-radiation within murine colorectal cancer (CRC) models [52]. Such an expression profile for miR-451a also correlated with a downregulation of Calcium-binding protein type 39 (CAB39) and BRCA2-interacting transcriptional repressor (EMSY), the latter two genes both acing as CRC biomarkers for poor prognosis [52]. The investigation carried out by Gao and colleagues elucidated the roles played by miR-888 in CRC from a total of 126 patients [53]. The results of this study, following Kaplan-Meier analysis and log-rank testing, revealed that miR-888 upregulation was collated to overall reduction in survival and disease-free survival from CRC, and such upregulation was also discovered within CRC biopsy tissues [53]. Consequently, this indicates the possible exploitation of miR-888 as a potential CRC prognosis biomarker [53].

miRNAs can also act as tumor suppressors within most human cancer models. This is also reflected in CRC, where a whole spectrum of miRNAs acts as regulators of the differing phenotype characteristics of CRC that make the tumor more aggressive and life threatening. The comprehensive review by Mizuno and colleagues gives a detailed representation of the Let-7 family of miRNAs that are typically downregulated in CRC cases [54]. In addition, the recent study conducted by Ke and colleagues elucidated miR-202-5p as a novel tumor-suppressor miRNA in CRC [55]. This investigation also concluded that miR-202-5p exerts such a tumor-suppressing role by direct regulation of a downstream oncogene (SWItch/Sucrose nonfermentable (SWI/SNF) related, matrix associated, actin dependent regulator of chromatin subfamily c member 1) that was previously established to be associated with CRC metastasis and tumor expansion properties [55]. More recent reports highlighting novel miRNA tumor suppressors in CRC include the study by Zhang and colleagues [56]. This particular study identified miR-1258 to be downregulated in CRC biopsies and representative cell lines, though its artificial upregulation led to an induction of cell cycle arrest at the G0/G1 phase in vitro and in vivo, together with inhibition of tumor cell proliferation through direct modulation of E2F Transcription Factor 8 (E2F8) [56].

## 7. miRNA Influences in Obesity-Linked Colorectal Cancer

The scientific reporting of miRNA activities involved in regulating physiological and molecular pathway links between obesity and colorectal cancer (CRC) is relatively scarce, though such observations are evermore on the increase (Table 1).

The initial findings by Olivio-Marston and colleagues in 2014 examined the possibility of diet-induced obesity to have a tumorigenic effect on CRC within the azoxymethane murine model [57]. In addition, calorie restriction (30% reduction of required daily calorific intake) was also applied on the same murine models to observe any possible converse relationships between diet and CRC [57]. Following a 10-week treatment, the murine group subjected to die-induced obesity had a 2.5-fold increase in CRC prevalence in comparison to the calorie-reduction murine group [57]. Furthermore, a total of 18 miRNAs were highly dysregulated within the diet-induces obesity murine group (eight upregulated and 10 downregulated) [57]. Following a shortlisting and consequent validation using Real Time Quantitative PCR (RT-qPCR) techniques, three miRNAs were validated to be upregulated in diet-induced obesity murines (miR-425, miR-196, and miR-155) and six miRNAs validated as downregulated within the same murine group (including miR-150, miR-351, miR-16, let-7, miR-34, and miR-138) [57].

In 2016, Meerson and Yehuda discovered a precise molecular interplay that placed miRNA activity as pivotal intermediates for the cross-communication between obesity and CRC [58]. This study consisted of treating colorectal cancer human cell lines (HCT-116, HT-29, DLD-1) with leptin and insulin, followed by miRNA expression profiling screens for approximately 800 miRNAs and consequent RT-qPCR validation of any shortlisted miRNA found to be dysregulated [58]. The validated results elucidated that miR-4443 was upregulated in two CRC cell lines after leptin/insulin exposure, with the DLD-1 cell line not demonstrating the same effect due to its lack of leptin receptor expression [58]. Consequent transfection of miR-4443 mimic within the HT-116 cell line resulted in a severe reduction in tumor invasion/proliferation properties [58]. Moreover, concomitant leptin treatment and miR-4443 transfection led to a severe downregulated expression level for Nuclear Receptor Coactivator 1 (NCOA1) and Tumor Necrosis Factor receptor-associated factor 4 (TRAF4), as both are confirmed target genes for miR-4443 and also previously established in orchestrating tumor metastasis properties [58]. Ultimately, this study comprehensively exposes the roles performed by miRNAs such as miR-4443, where in this case miR-4443 acts as a tumor-suppressor miRNA in CRC due to leptin/insulin signaling [52]. However, obesity-induced leptin and/or insulin resistance could thwart such tumor-suppressing activity by miR-4443 on TRAF4/NCOA1 and lead to potentiating the risks for CRC clinical presentation in obese individuals [58].

Continuing in this line of research, the investigations carried out by Tie and colleagues in 2017 revealed further details on the level of miRNA interplay with hypercholesterolemia and CRC [59]. Employment of two murine models (ApoE-/- and C57BL/6) allowed for analysis of any correlations between a high cholesterol diet and CRC incidence in murines [59]. Following the induction of the C57BL/6 murine group to a high cholesterol diet, azoxymethane was administered to both groups for induction of CRC [59]. The results of this phase of the study revealed that incidences of CRC were almost twofold higher in the C57BL/6 murine group that was fed a high cholesterol diet [59]. Further analyses revealed that such hypercholesterolemia led to oxidative stress-related triggering of a miR-101c expression level, consequently inducing a direct downregulation of Tet1 within hematopoietic stem cells and ultimately reducing the expression levels of multiple genes associated with natural killer T cells (NKT) and gamma-delta T-cell development [59]. This study therefore represents an excellent portrayal of how, in this case, an obesity comorbidity status (hypercholesterolemia) led to specific miRNA dysregulations (miR-101c) that ultimately led to a reduction in the efficiency of immune-surveillance against CRC through epitranscriptomic modifications of T-cell differentiation genes [59].

Another scientific report of importance was the recent publication by Motawi and colleagues in 2017 [54]. This study mainly focused on the effects of the dysregulated expression of peroxisome proliferator-activated receptor gamma (PPAR-*γ*) in obesity [60]. Blood samples were collected from four CRC patient/control groups (34 CRC obese, 36 CRC lean, 22 obese control, and 24 lean control), with consequent serum analysis for circulating miRNA profiling and including peripheral blood mononuclear cell (PBMC) PPAR-*γ* expression level and degree of its promoter methylation [60]. The results of this study elucidated a major upregulated expression level for miR-27b, miR-130b, and miR-138 within CRC and obese patients [60]. Furthermore, PPAR-*γ* expression was also found to be downregulated within the same patient groups, consequently inferring the miRNA interplay to lead to increased CRC risks within patients through a downregulatory action by such a miRNA combination on PPAR-*γ* expression [60].

## 8. Perspective and Future Directions

There are already a number of reputable industry leaders in miRNA therapies such as miRNA therapeutics, miRagen Therapeutics, Santaris pharma, or Regulus Therapeutics. They are currently entering clinical trials, using miRNAs for the treatment of several diseases, including cancer. The purpose of miRNA therapies is mainly using miRNAs that normally downregulate several target-oncogene, as possible drugs. The loss of function of these miRNAs might alter the expression of target-oncogene. Therefore, it could initiate tumor formation. These miRNAs should function as an inhibitor that antagonize (by sequence complementarity) the upregulated oncomiR in order to artificially lower the oncomiR's expression level to “normal” levels. Alternatively, the patient can be treated with miRNA mimics in order to artificially increase the expression levels of downregulated tumor-suppressor miRNAs. In both cases, a bespoke drug delivery system is employed to assure safe passage of such miRNA mimics/inhibitors through the bloodstream and allow uptake at the site of action [61].

Approximately 20 clinical trials against cancer have currently been conducted using miRNA-based therapeutics [62]. miRNAs are involved in many critical processes such as tumor initiation, progression, and invasion. All of the recent published evidence suggests that inhibition of some overexpressed oncogenic miRNAs could provide a robust strategy for cancer therapy. Beg and colleagues in 2013 designed the first miRNA replacement therapy on human clinical trials for the treatment of advanced or metastatic liver cancer. miR-34 has been presented as a powerful tumor suppressor by the biopharmaceutical company “miRNA Therapeutics”, as MRX34, a liposomal miR-34a mimic. This drug can be used in a wide variety of cancers. The MRX34 phase 1 is now being conducted as a clinical trial in liver cancers. However, this clinical trial was stopped in 2016 due to multiple immune adverse events [63].

The gene-miRNA-diet interaction could be a promising target to consider miRNA as drug therapies in the obesity-related cancer axis. A study conducted in prostate cancer cell lines found that genistein (isoflavone) was able to upregulate ADP–Ribosylarginine-Hydrolase (ARH1), a suppressor tumor, by downregulating miR-221 and miR-222 [64]. Curcumin inhibits miR-21, invasion, and metastasis in colorectal cancer [65]. Curcumin also induces the apoptosis of gastric cancer cell lines through upregulation of miR-33b [66]. miR-27a, a miRNA released from adipose tissue cells, promotes the proliferation of liver cancer [67]. There are several evidences on the interaction between genes, diet, obesity, and cancer. However, more studies in obesity-linked colorectal cancer on interaction-communication via miRNA must be considered to increase interest in developing novel miRNA-based theragnostic strategies to counteract and/or mitigate the potentially life-threatening outcomes of untreated colorectal tumors within afflicted obese patients and provide a possible target for diagnosis and therapy.

Enhancing awareness among the scientific community about the prospect of miRNA influences is crucial to consider miRNA therapies in obesity-linked colorectal cancer tumorigenesis and its clinical progression. Research articles pertaining to the identification and validation of such crucial miRNA biomarkers in this bespoke niche of cancer patients are still very low and, consequently, it is necessary to increase the spotlight and eventually increase the level of global research efforts for discovering novel miRNA influences of obesity-linked colorectal cancer. Ultimately, additional research will eventually lead to novel miRNA-based theragnostic tools that can benefit the cancer patient within the clinical setting in the not-too-distant future. Currently, we have enough knowledge about the role of miRNA as a diagnostic tool, but we must gather all the efforts to turn them into a therapeutic tool.

Although miRNA-based therapeutics and diagnostics are still in their infancy, the degree of bespoke therapeutic efficacy and enhanced patient risk-stratification precision offered by such technology will certainly grow to the point where novel diagnostic kits and drug treatments for the treatment of all conditions influenced by miRNA dysregulations will become the mainstay clinical tools of the consultant oncologist by the end of the next decade. Recent technological advances could allow a high performance to obtain the specific profiles of miRNA (fingerprint) at a very precise level, in a cost-effective manner in the diagnosis, treatment, and monitoring of obesity and cancer. The low cost of data generating could lead us to the Big Data Era. The availability of these large datasets provides unprecedented opportunities but also poses new challenges for data mining and more accurate analysis.

## 9. Conclusions

Even though the current level of evidence is at its infancy, epigenetics mechanism involved to regulatory elements influences within all aspects of human cell physiology pathways can never be underestimated. In this review, we aimed to shed light on merely how epigenetics modifications could induce molecular orchestration that interplays between obesity and colorectal cancer tumorigenesis and development. Within the short-term future, the authors are highly confident that further evidence will be revealed by the global scientific community to further confirm the paramount roles played by DNA methylation and miRNAs within the obese individual, both for inducing comorbidities and severe disease pathologies and for possible prophylaxis and treatment measures.

## Figures and Tables

**Table 1:** List of clinically significant miRNA-driven interplays between obesity and colorectal cancer development/disease progression.

miRNA/s involved	Functional role of miRNA/s (when up-regulated)	Affected pathways and/or gene/s	References
miR-425	Detrimental	Obesity development	[57]
miR-196			
miR-155			
miR-150			
miR-351			
miR-16			
let-7	Detrimental	Obesity development	[57]
miR-34			
miR-138			
miR-4443	Detrimental	NCOA1 and TRAF4	[58]
miR-101c	Detrimental	Tet1	[59]
miR-27b			
miR-130b	Detrimental	PPAR-*γ*	[60]
miR-138			
